# Receptors and effects of gut hormones in three osteoblastic cell lines

**DOI:** 10.1186/1472-6793-11-12

**Published:** 2011-07-29

**Authors:** Elda L Pacheco-Pantoja, Lakshminarayan R Ranganath, James A Gallagher, Peter JM Wilson, William D Fraser

**Affiliations:** 1 Escuela de Medicina, Universidad Anáhuac Mayab, Km 15.5 Carr Merida-Progreso, 97310, Mérida, Yucatán, México; 2 Department of Clinical Biochemistry and Metabolic Medicine, Royal Liverpool University Hospital, Prescot Street, L78XP, Liverpool, UK; 3 Department of Human Anatomy and Cell Biology, University of Liverpool, Sherrington Buildings, Ashton Street, L69 3GE, Liverpool, UK; 4 Musculoskeletal Biology Department, University of Liverpool, 4th Floor Duncan Building Daulby Street, L69 3GA, Liverpool, UK

## Abstract

**Background:**

In recent years the interest on the relationship of gut hormones to bone processes has increased and represents one of the most interesting aspects in skeletal research. The proportion of bone mass to soft tissue is a relationship that seems to be controlled by delicate and subtle regulations that imply "cross-talks" between the nutrient intake and tissues like fat. Thus, recognition of the mechanisms that integrate a gastrointestinal-fat-bone axis and its application to several aspects of human health is vital for improving treatments related to bone diseases. This work analysed the effects of gut hormones in cell cultures of three osteoblastic cell lines which represent different stages in osteoblastic development. Also, this is the first time that there is a report on the direct effects of glucagon-like peptide 2, and obestatin on osteoblast-like cells.

**Methods:**

mRNA expression levels of five gut hormone receptors (glucose-dependent insulinotropic peptide [GIP], glucagon-like peptide 1 [GLP-1], glucagon-like peptide 2 [GLP-2], ghrelin [GHR] and obestatin [OB]) were analysed in three osteoblastic cell lines (Saos-2, TE-85 and MG-63) showing different stages of osteoblast development using reverse transcription and real time polymerase chain reaction. The responses to the gut peptides were studied using assays for cell viability, and biochemical bone markers: alkaline phosphatase (ALP), procollagen type 1 amino-terminal propeptides (P1NP), and osteocalcin production.

**Results:**

The gut hormone receptor mRNA displayed the highest levels for GIP in Saos-2 and the lowest levels in MG-63, whereas GHR and GPR39 (the putative obestatin receptor) expression was higher in TE-85 and MG-63 and lower in Saos-2. GLP-1 and GLP-2 were expressed only in MG-63 and TE-85. Treatment of gut hormones to cell lines showed differential responses: higher levels in cell viability in Saos-2 after GIP, in TE-85 and MG-63 after GLP-1, GLP-2, ghrelin and obestatin. ALP showed higher levels in Saos-2 after GIP, GHR and OB and in TE-85 after GHR. P1NP showed higher levels after GIP and OB in Saos-2. Decreased levels of P1NP were observed in TE-85 and MG-63 after GLP-1, GLP-2 and OB. MG-63 showed opposite responses in osteocalcin levels after GLP-2.

**Conclusions:**

These results suggest that osteoblast activity modulation varies according to different development stage under different nutrition related-peptides.

## Background

Bone is a tissue subjected to constant forces and remodelling, requiring a satisfactory nutrient intake to maintain bone mass. It has previously been suggested that there is a direct association between food intake and bone turnover as assessed by biochemical markers of bone resorption and formation [1,2]. Some observations indicate that there are other mechanisms regulating the interaction between nutrition and bone homeostasis, in addition to those well studied processes involving vitamin D or parathyroid hormone (PTH) [3].

Among the alternative regulatory mechanisms, hormones produced in the gastro-intestinal tract may play an essential role. These gastro-entero-pancreatic hormones are important gastrointestinal-releasing hormones involved in the regulation of postprandial nutrient homeostasis [4]. The interest in gut hormones and their relationship to bone metabolism has been increasing, presenting the possibility of alternative treatments and/or targets against bone degeneration. The connection between gut hormones and bone has been cited as an entero-osseous-axis [5] to resemble the term entero-insular axis, which refers to the signalling pathways between the gut and pancreatic islets that enhance the insulin response to absorbed nutrients [6].

The present study is focused on five of these gut hormones and their effects on osteoblast-like cell lines: two incretin hormones glucose-dependent insulinotropic peptide (GIP), glucagon-like peptide 1 (GLP-1), the related glucagon-like peptide 2 (GLP-2), and the two preproghrelin gene products, ghrelin (GHR) and obestatin (OB).

Previous studies have shown that GIP is able to increase collagen type I expression and alkaline phosphatase (ALP) activity in osteosarcoma cell lines (Saos-2, MG-63, ROS 17/2.8) [7], and to a certain extent has a protective effect on osteoblast apoptosis [8]. The role of GIP in modulation of bone turnover has been studied using knockout mice models, and the results showed less bone formation, smaller bone size, lower bone mass alterations in bone microarchitecture and biomechanical properties, in GIP receptor knockout mice [9]. Another study has shown that GIP inhibited resorptive activity of osteoclasts [10].

Reports of GLP-1 effects on bone metabolism are limited and, equivocal. Although, receptors for GLP-1 had not been demonstrated in human osteoblasts it has been suggested that these receptors could be vital for some processes in bone turnover, especially those related to resorption [11,12]. Moreover, a functional receptor for GLP-1 using a pathway non-dependant of cAMP has been reported in a murine osteoblastic cell line [13]. Among other actions, GLP-1 has an important role in apoptosis, differentiation and intracellular effects on calcium in human pancreatic islet cells [14,15].

A number of studies have demonstrated a clearer relationship between GLP-2 and bone metabolism. A study showed that patients, with small-bowel resection and colon resection receiving a subcutaneous dose of GLP-2 had positive effects on their bone mineral density (BMD) but the levels of the bone turnover markers did not clarify on the involved mechanisms [16].

Henriksen *et al *[17] studied postmenopausal women in randomized placebo-controlled studies and showed that GLP-2 transiently suppressed the nocturnal rise in β-CTX compared to control. In the same report [17] a dose-dependent effect of GLP-2 on bone formation was observed. In both cases, there were significant reductions in β-CTX. In addition, the authors measured osteocalcin and this was increased compared to placebo, indicating a dose-dependent effect of GLP-2 on bone formation.

Ghrelin is an endogenous ligand for GHS-R, which has been purified and characterized, and acts as an agonist for an orphan receptor [18]. The data suggest that ghrelin signalling may be essential for normal growth in humans and some authors have reported a significant stimulation of osteoblast proliferation and an increased ALP activity in osteoblasts in response to ghrelin [19,20], and a suspected direct regulation of bone formation [21].

Based on data provided by bioinformatics, a ghrelin-associated peptide was identified and called obestatin. In *in vitro *observations this peptide, unlike ghrelin, did not increase growth hormone secretion by cultured rat pituitary cells [22], however other investigations have shown that obestatin is capable of growth hormone modulation in growth cells from a rat somatotroph tumour [23].

To date, no available information on the effects of obestatin on bone metabolism is available. Its receptor is believed to be an orphan receptor G-coupled protein (GPR-39) [22], although some discrepancies have been reported, in which GPR39 could not be activated by obestatin but by high concentrations of zinc ions [24,25], although more recent studies demonstrated that transfected cells with a plasmid encoding GPR39 exhibited high -affinity binding to an analogue of obestatin (monoiodobestatin) [26].

This study investigated the expression of five gut hormone receptors (GIP-R, GLP-1R, GLP-2R, ghrelin receptor [GHS-R] and GPR39) when RNA from three osteoblastic cell lines was extracted, reverse transcribed and analysed using real time amplification. In addition, the expression of collagen 1 alpha 2 (COL1A2), ALP and OPG was included to profile some features of these cell lines. The functional responses to increasing concentrations of these hormones, on all three osteoblast cell lines are reported in terms of cell viability levels, ALP activity, N-terminal propeptide of type I procollagen (P1NP) and osteocalcin determinations in culture supernatants. The cell lines used in the present model exhibits the following background:

MG-63. It is a cell line derived from an osteosarcoma. They are considered to display features of an undifferentiated early osteoblast phenotype. These cells produce high amounts of interferon, and provide suitable models for studying comparable integrin subunit expression and osteocalcin production to those in primary bone cells, but they are not good models for proliferation (higher rate), ALP (poor production) or osteonectin production [27,28].

TE-85. Referred to as HOS or TE-85, they were derived from the sarcoma of a female Caucasian. These cells have a higher production of ALP than MG-63, but a low synthesis of osteocalcin. As MG-63, they are suitable models to study integrins [27].

Saos-2. Derived from the osteosarcoma of a female Caucasian. These cells are excellent producers of ALP, but osteocalcin is poorly produced in these cells. They are highly sensitive to PTH stimulation [29]

## Methods

### Cell culture of the osteoblastic-like cell lines

Three cell lines were used: Saos-2, TE-85, and MG-63. All of them were grown in DMEM (Sigma, UK) supplemented with 10% fetal calf serum (FCS) (Biosera, UK), 50 U/ml penicillin, 50 μg/mL streptomycin. For the study of the receptors cells were grown near confluence in 9-cm Petri dishes. For the functional studies, the cells from the second passage were seeded for 24 hours in 24-well plates, and serum deprived for 24 hours. After this time the medium was changed for modified medium supplemented with gut hormones, in different concentrations depending on the experiments to be performed. After the exposure to peptides, the assays were performed for cell viability, ALP, P1NP and osteocalcin secretion. Cells growing in medium without any of the gut peptides were used as controls. The experiments were set individually per cell line. The arrangement of wells was randomized within the plates to avoid "edge-effects". The gut peptides used were human GIP, GLP-1 (7-36) amide, GLP-2 purchased from PolyPeptide Laboratories GmbH, Wolfenbüttel, Germany. Human ghrelin (octanoyl) and obestatin Gly-Lys were obtained from Phoenix Europe GmbH (Karlsruhe, Germany).

### Reverse transcriptase polymerase chain reaction (RT-PCR)

The presence of expressed receptors was detected by extraction and reverse transcription of RNA obtained from confluent cell cultures. Briefly, the standard protocol is as follows: Tri reagent-chloroform was used to extract mRNA and reverse transcribed, with Superscript II (Invitrogen, UK) to obtain the cDNA first strand. PCR screening was performed in a MJ Research thermal (Labcare, UK) cycler using a 10 μl reaction volume containing cDNA, dNTPs, PCR buffer with added MgCl_2_, the appropriate oligonucleotide primers of each receptor gene and Hot Start^® ^taq polymerase. The protocol used the following cycles: initial denaturation for 15 min, followed 40 cycles of denaturation (20 sec), annealing (40 sec), and extension (40 sec), and a final extension cycle (5 min). The reaction products were separated on a 1% agarose gel in Tris-borate-EDTA buffer, containing SYBR safe (Invitrogen), and were run at 100 volts.

### Real-time RT-PCR quantitation (RT-qPCR) of gene expression

In order to compare the rates of the gene relative expression, RT-qPCR was performed and monitored using iCycler iQ (Bio-Rad, UK) following the protocol recommended by the manufacturer. cDNA samples were analyzed both for the genes of interest (gut hormone receptors) and the reference gene (β-actin). The cycling program was as follows: denaturing at 95°C for 3 min followed by 40 cycles of annealing and denaturing for 30 s. Reactions were done in triplicate on a 96-well plate in two different runs. Cycle threshold (Ct) values were obtained and efficiency parameter was calculated with LinRegPCR software for each well. Both parameters were used to calculate the relative rate expression for each gene of interest using β-actin as a normalizer [30].

The primer oligonucleotide sequences are shown in Table 1.

**Table 1:** Details of primer sequences used in qPCR analysis

Gene of interest	Accesion numbers	Sequence of primers	Product size	Annealing temp (°C)
COL1A2	NM_000089	5' GGCACTCCAGGTCCTCAG 3' 5' CCACAGCACCAGCAACAC 3'	100	60
ALP	NM_000478	5' GCTGAACAGGAACAACGTGA3' 5' TCAATTCTGCCTCCTTCCAC 3'	117	60
OPG	NM_002546	5'GCAGCGGCACATTGGACATG 5'AGGATCTGGTCACTGGGTTTGC3'	135	60
β-ACTIN	NM_001101	5' GGACCTGACTGACTACCTC 3' 5' GCCATCTCTTGCTCGAAG 3'	135	60
GIPr	NM_000164	5' GACCAAAGGCTCATCTTGGA 3' 5' ATGTAGCCGCCTGAACAAAC 3'	114	60
GLP-1r	NM_002062	5' TGGACCAGGAACTCCAACAT 3' 5' TTTGGATACCACGATGCAGA 3'	114	62.5
GLP-2r	NM_004246	5' TTCCTTTATTGGGCGTTCA 3' 5' CTCTCCATTGGCAAAACCA 3'	155	60
GHSr 1a	NM_198407	5' GCACTCTTCGTGGTGGGCAT 3' 5' GATGAGCAGATCGGAGAAGG 3'	123	60.5
GPR39	NM_001508	5' GCTCATGAAAAGCCAGAAGG 3' 5' CATGATCCTCCGAATCTGGT 3'	172	60

### Viability assay

The assay for viability was performed, using a fluorogenic, cell permeant, peptide substrate (glycil-phenylalanyl-amino-fluorocoumarin, [GFAFC], Promega). Cells were seeded into 96-well black wall plate (clear bottom), at a density of 10,000 cells per well in 100 μL medium, serum deprived 24 h, and treated with the gut peptides at final concentrations from 10^-12 ^to 10^-9^, using 10 replicates per concentration per peptide. The viable cell protease marker assays were conducted using GF-AFC at 10^-4^M final concentration (as recommended by the manufacturer). Readings for relative units of fluorescence were obtained using the Cytofluor series 4000 equipment (Applied Biosystems), filters for excitation ~400 nm, and emission ~505 nm (photomultiplier gain factor 40).

### ALP activity

ALP was measured in culture supernatants using a procedure which involves a colorimetric assay using p-nitrophenyl phosphate as substrate, and measuring spectrofotometrically the p-nitrophenol released at 405 nm with the absorbance being proportional to the ALP activity. Cells were seeded onto 24 well plates and cultures were treated with the gut peptides at final concentrations ranging from 10^-12 ^to 10^-9 ^M and supernatants were collected for further ALP assay. The determinations were performed using 10 replicates each time. All the treatments were compared against control wells (cell culture with ordinary DMEM without hormone peptides).

### P1NP assay

The assay was performed using an electrochemiluminescence immunoassay (ECLIA) in an Elecsys Immunoassay System (Roche) and measures both monomeric and trimeric forms of P1NP. Cells were treated with gut peptides at two final concentrations of 10^-11 ^and 10^-9 ^M, five replicates were used per treatment, and supernatants were collected to perform the determinations.

### Osteocalcin assay

This determination was performed using ECLIA, and measures N-terminal midfragment osteocalcin. Cells were treated as in the latter assay: gut peptides were added at two final concentrations of 10^-11 ^and 10^-9 ^M, five replicates were used per treatment, and supernatants were collected to perform the determinations.

### Statistical analysis

Data obtained in viability, ALP, P1NP, and osteocalcin assays were analyzed with SPSS software, using the test of Levene to assess the equality of variances. The significance was set at P-values less than 0.05. Student's t-test was used to compare individually the means of each treatment. Results are shown as the fold or percentage changes plus-minus standard error of the mean (SEM)

## Results

### Expression of gut hormone receptors detected by PCR

mRNAs were extracted from the three osteoblastic cell lines (MG-63, TE-85, Saos-2), and treated with DNAse to remove traces of genomic DNA contamination and followed by reverse transcription. The mRNA level was quantitated by RT-qPCR using designed primers for all the genes of interest (two bone markers and five gut hormone receptors). β-actin was used as the housekeeping gene to standardise between samples.

**Figure 1:**
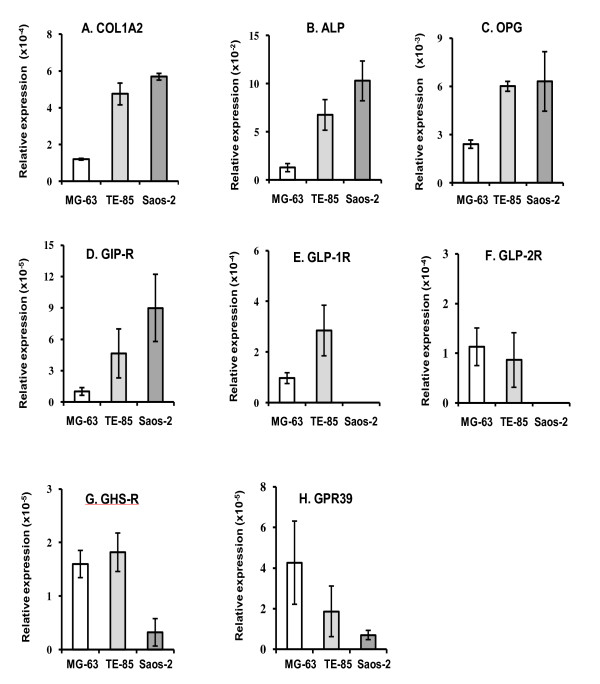
**COL1A2, ALP, OPG and gut hormone expression profiles**. After extraction and reverse transcription, mRNA levels were analysed. Results are expressed as means of at least six replicates per cell line ±SEM. β-actin was used as the housekeeping gene to normalize the levels of mRNA expression. In all the cases these determinations showed highest expression in Saos-2 and the lowest rate was for MG-63. Water was used as non-template control. COL1A2, ALP and OPG had their highest expression in Saos-2, and the lowest in MG-63. GIP-R, GHS-R, and GPR39 mRNA expression was found in the three osteoblastic cell lines. GLP-1R and GLP-2R expression was observed only in MG-63 and TE-85.

The RT-qPCR procedure was performed to study mRNA expression levels for COL1A2, ALP, OPG and the gut hormone receptors. The results showed that COL1A2, ALP and OPG had the highest expression in Saos-2, and the lowest in MG-63. COL1A2 mRNA levels demonstrated rates of 4 and 4.7 times in TE-85 and Saos-2, respectively, over MG-63 (Figure 1A). ALP, showed rates of 5.2 and 8 times in TE-85 and Saos-2 over MG-63 (Figure 1B). OPG was also best expressed in TE-85 and Saos-2, with rates of 2.5 and 2.6 respectively over MG-63 (Figure 1C). From these determinations a pattern was observed, in which Saos-2 is identified as the cell line being the most mature or differentiated and MG-63 as the least differentiated. TE-85 may have a level of maturity somewhere between MG-63 and Saos-2.

mRNA relative expression for each gut hormone receptor genes, showed that GIP-R was in direct relation with the differentiation degree, thus the most mature cell line, Saos-2, had the greatest level of expression and it was decreasing according to the maturity level (Figure 1D). The rates of GIP-R expression for Saos-2 were about 9 times greater than MG-63 and 2 times those expressed in TE-85.

In the case of GLP-1R (Figure 1E) and GLP-2R (Figure 1F), their expression was observed in TE-85 and MG-63, but it could not be confirmed in Saos-2. GLP-1R in TE-85 displayed higher levels of mRNA level, whereas GLP-2R had a higher level in MG-63, but no major differences were observed between TE-85 and MG-63. GHS-R showed greater expression in the least mature cell lines, 5 fold higher than the lower expression in Saos-2 (Figure 1G). GPR-39 followed an inverse relation with the maturity degree, with the highest expression in MG-63 and the lowest expression in Saos-2 (Figure 1H). The specificity of the RT-qPCR was further confirmed with the analysis of the melting curves, set after the standardised 40 cycles of amplification.

**Figure 2:**
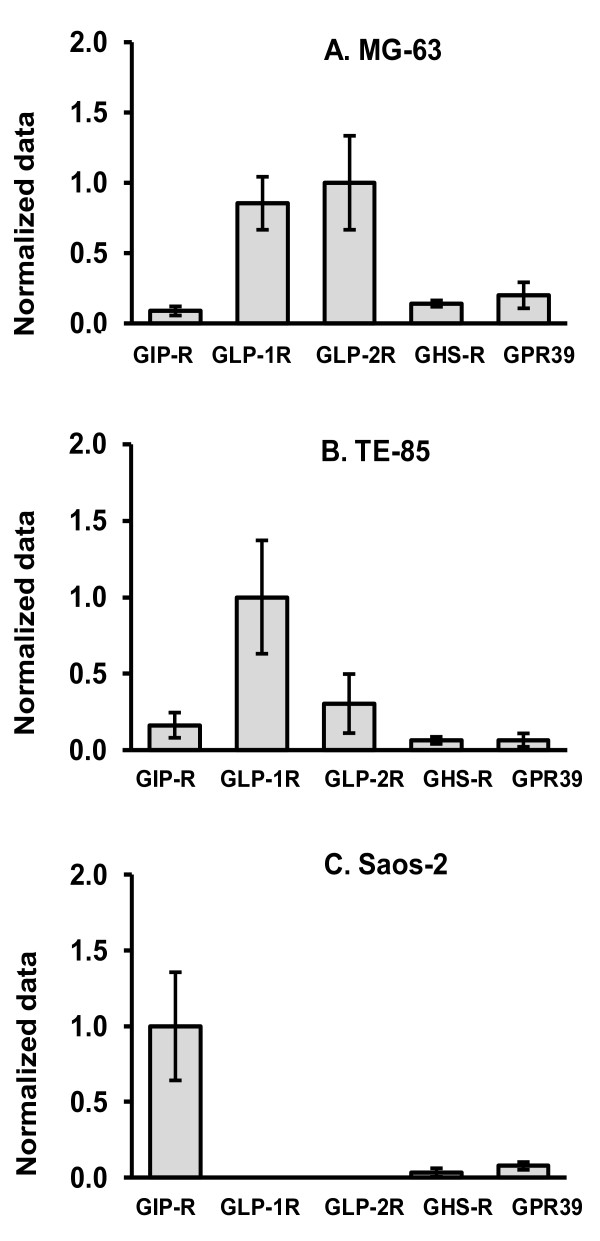
**Expression of the gut hormone receptors in cell lines**. After normalizing mRNA levels to β-actin, they were re-normalized again to the gene which presented the highest rate in each cell line, in order to display a profile for each cell line. This normalisation showed that GLP-1r and GLP-2r have a higher expression in MG-63 (A); GLP-1r is predominantly in TE-85 (B); GIPr is the most important in Saos-2 (C); and GHSr and GPR39 have the lowest expression in the three cell lines (A,B,C). Data are presented as the means of normalized data ± SEM.

Figure 2 summarises the findings for the receptors per cell line. After calculations for the level of expression using β-actin, data were normalized again using the highest point for each cell line, and assigning it the arbitrary value of 1, to find the profile per cell line. This analysis shows that GLP-1R and GLP-2R were most expressed in MG-63 and TE-85, but GIP-R was preferentially expressed in Saos-2, leaving GHS-R and GPR39 with the lowest level of expression for the three cell lines.

### Cell viability, ALP, P1NP and osteocalcin assays Viability assay

**Figure 3:**
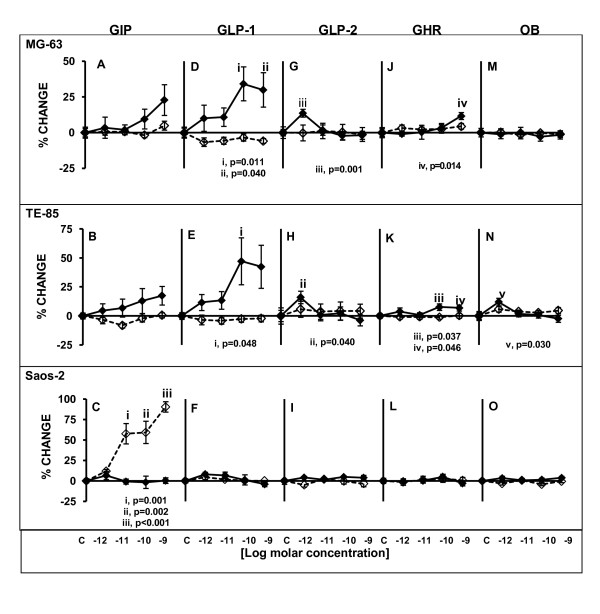
**Cell viability levels after treatments with gut hormones in three osteoblastic cell lines**. Cells were treated for 48 or 120 h with the gut peptides at indicated molar concentrations. Straight lines solid diamond markers designate 48 h treatment; dashed lines emptied diamond markers indicate 120 h treatment. In this assay, GIP induced statistically significant increases in Saos-2 after 120 h in a dose-dependent manner (C), GLP-1 (D,E), GLP-2 (G, H)) and GHR (J, K) caused increases in MG-63 and TE-85, and the only positive responses for OB were observed in TE-85 (N). Results are the average of percentage of change in relation to controls; n = 10. ± SEM.

In general, the viability test showed unaffected or significant higher changes levels of cell viability after exposure to peptides. GIP did not cause any significant changes in MG-63 or TE-85 viability, although the trend was on the increase at 48 h exposure (Figure 3A, B). Conversely, in Saos-2, no changes were observed after 48 h, but a statistically significant dose-dependent increase was observed after 120 h of exposure to the peptide (10^-11^M p = 0.001, 10^-10^M p = 0.002, 10^-9^M p < 0.001) (Figure 3C).

GLP-1 caused significant increases in cell viability in MG-63 at 10^-10 ^M (p = 0.011) and 10^-9 ^M (p = 0.040) (Figure 3D). In TE-85 significant changes were observed at 10^-10 ^M (p = 0.048) (Figure 3E). In both cases the significant responses were registered at 48 h. Saos-2 did not exhibit any changes after exposure to this peptide (Figure 3F). In the case of GLP-2, significant changes were observed in the viability of MG-63 (p = 0.001) (Figure 3G) and TE-85 (p = 0.040) (Figure 3H), with increases after 48 h, at 10^-12^M but no other changes were observed at higher concentrations or extended treatment times. Saos-2 did not display any changes in response to this peptide in the viability test (Figure 3I).

GHR induced a significant increase in MG-63 (Figure 3J) viability at its highest concentration (p = 0.014) after 48 h of exposure. Also, TE-85 (Figure 3K) displayed significant increases at 10^-10^M (p = 0.037) and 10^-9^M (p = 0.046) after 48 h in the viability test, but no changes were observed after 120 h. Saos-2, did not exhibit any change (Figure 3L). OB caused significant changes increasing the viability in TE-85 (Figure 3N) at 10^-12^M after 48 h (p = 0.030). MG-63 and Saos-2 (Figure 3M, O) did not show changes in viability after exposure to this peptide.

### ALP production

In terms of significant ALP activity changes in supernatants induced by the presence of gut hormones, GIP, GHR an OB caused significant increases in Saos-2 and TE-85 (only with GHR).

**Figure 4:**
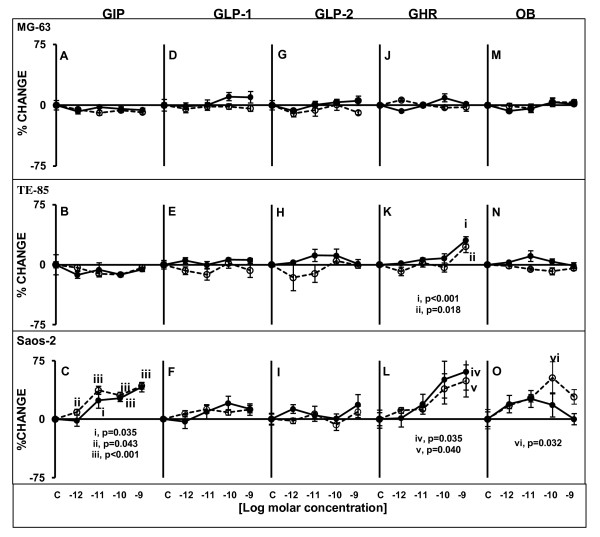
**ALP levels after treatments with gut hormones in three osteoblastic cell lines**. Cells were treated for 48 or 120 h with modified medium containing the gut peptides at indicated molar concentrations. Straight lines circle solid markers designate 48 h treatment; dashed lines emptied circle markers indicate 120 h treatment. Only Saos-2 and TE-85 displayed statistically significant responses to gut peptides in ALP production. Saos-2 responded to GIP (C), GHR (L) and OB (O). TE-85 was stimulated only by GHR (K). MG-63 did not show any significant changes. Results are the average of percentage of change in relation to controls; n = 10, ± SEM.

GIP did not have any effect on ALP production by MG-63 (Figure 4A) or TE-85 (Figure 4B), but it caused significant increases that were dose-dependent in ALP production by Saos-2 (Figure 4C) after 48 and 120 h. The significant changes observed at 48 h were at 10^-11^M (p = 0.035), 10^-10^M (p < 0.001) and 10^-9^M (p < 0.001); for 120 h the significant increases were observed starting with the lowest concentration: 10^-12^M (p = 0.043), 10^-11^M (p < 0.001), 10^-10^M (p < 0.001) and 10^-9^M (p < 0.001).

No effects were observed after exposure to GLP-1 and GLP-2 in any of the cell lines (Figures 4D-1)

GHR did not cause any changes in ALP production by MG-63 (Figure 4J). However, the presence of this peptide caused significant increases in ALP production by TE-85 (Figure 4K) and Saos-2 (Figure 4L) when the highest concentration (10^-9^M) was present in the culture medium at the two time points investigated. For TE-85, p < 0.001 at 48 h and p = 0.018 at 120 h. In the case of Saos-2, p = 0.035 at 48 h, and p = 0.040 at 120 h.

Even though OB did not cause any significant increases in ALP activity in MG-63 (Figure 4M) and TE-85 (Figure 4N), Saos-2 displayed a significant response after 120 h at 10^-10^M (p = 0.032), and a bell-shaped like curve was observed after 48 h (Figure 4O).

### P1NP secretion

**Figure 5:**
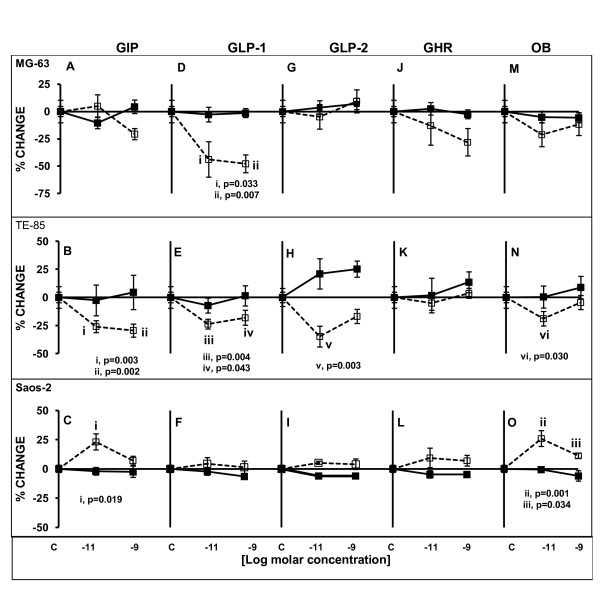
**P1NP levels after treatments with gut hormones in three osteoblastic cell lines**. Cells were serum deprived and treated for 48 or 120 h with the gut peptides at indicated molar concentrations. Straight lines solid square markers designate 48 h treatment; dashed lines emptied square markers indicate 120 h treatment. Significant higher production of P1NP after 120 h exposure to GIP and OB were observed only in Saos-2 (C, O). The least mature cell lines, MG-63 and TE-85 showed decreased P1NP secretion in response to GIP (A), GLP-1 (D, E), GLP-2 (H) and OB (N) stimulation after 120 h exposure. In all the cases the most significant changes were observed with the lowest concentration of peptide. Results are the average of percentage of change in relation to controls; n = 5. ± SEM.

GIP did not cause any significant changes in MG-63 in P1NP production (Figure 5A). In TE-85 a significant decrease in P1NP secretion was observed after 120 h exposure with the two concentrations tested, 10^-11^M and 10^-9^M, (p = 0.003 and p = 0.002 respectively) (Figure 5B). Conversely, GIP significantly increased P1NP secretion by Saos-2 cells at 10^-11^M after 120 h (p = 0.019), but no differences were observed at the highest concentration at any time (Figure 5C).

GLP-1 induced in MG-63 a significant decrease in P1NP secretion after 120 h at 10^-11^M and 10^-9^M (p = 0.033 and p = 0.007, respectively) (Figure 5D). Also, TE-85 exhibited significant decreases after 120 h, at both concentrations (p = 0.004, p = 0.043) (Figure 5E). Saos-2 did not display any significant differences at any point (Figure 5F).

GLP-2 only caused significant changes in P1NP secretion by TE-85 cells after 120 h when the concentration was 10^-11^M (p = 0.003) (Figure 5H). No statistically significant alterations were observed in MG-63 (Figure 5G) or Saos-2 cultures (Figure 5I) when they were treated with GLP-2.

In the case of GHR, no significant changes were observed after treatment with this peptide in any of the cell lines (Figure 5J-L).

OB caused opposite actions in two of the cell lines: in TE-85 there was a significant decrease after 120 h at 10^-11^M (p = 0.030) (Figure 5N), but in Saos-2 the levels of P1NP were increased above 25% of change from control at 10^-11^M (p = 0.001) and at 10^-9^M (p = 0.034) after 120 h, however the latter increase was smaller than the former (Figure 5O). MG-63 registered a decrease but this was not significant (Figure 5M).

### Osteocalcin secretion

The results for this assay corresponded only to those obtained from MG-63 experiments. The reason was that Saos-2 and TE-85 did not produce significant amounts of this protein, and there was a minimum difference when compared against blanks (medium from wells containing no cells). However, OC from MG-63 cultures were significantly different from the blanks making MG-63 the only cell line in this study that could be used as a model to investigate the production of this protein *in vitro*.

**Figure 6:**
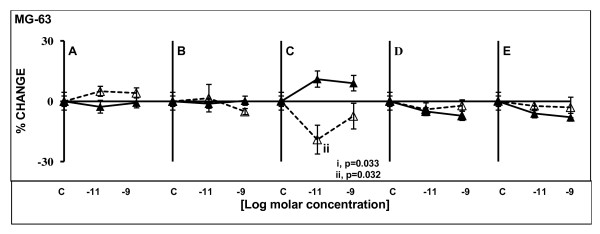
**Osteocalcin levels after treatments with gut hormones in MG-63 osteoblastic cell lines**. Cells were serum deprived and treated for 48 or 120 h with the gut peptides at indicated molar concentrations. Straight lines solid triangle markers designate 48 h treatment; dashed lines empty triangle markers indicate 120 h treatment. Only GLP-2 (C) caused significant changes in OC production in MG-63, with increased secretion levels after short exposure, and decreased production after longer exposure. Results are the average of percentage of change in relation to controls; n = 5. ± SEM.

No statistically significant differences were found after stimulation with GIP, GLP-1, GHR and OB (Figure 6A, B, D, and 6E). However, GLP-2 caused different patterns in OC production depending upon the time of exposure: after 48 h a significant increase was observed with the lowest concentration of the hormone (p = 0.033), and the difference was not significant with the high concentration but a plateau was established. After 120 h exposure, a significant decrease was observed at low concentration (p = 0.032) and no significant changes were observed with the high concentration, but the percentage of change was below the control (Figure 6C).

## Discussion

This work presents a study of the receptors for the gut hormones GIP, GLP-1, GLP-2, GHR and OB, in three osteoblastic cell lines. The chosen *in vitro *model represents different stages of osteoblastic development and has been valuable in analysing the patterns of expression of the genes of interest in different levels of osteoblastic maturity. Although investigation of cell lines may differ in some responses from naturally occurring primary cells the use of cell lines represents a suitable model to study determined characteristics in a specific stage of osteoblast maturity as an initial observation.

COL1A2, ALP and OPG mRNA expression were used as bone markers that are correlated with osteoblastic differentiation. It has been shown that type 1 collagen mRNA signal increases according to the degree of differentiation in cultured rat calvaria cells [31]. ALP is associated with the phase of differentiation and stabilisation of mineralised matrix [32]; OPG mRNA expression has been found to be increased during matrix production and maturation in rat calvaria cultures [33]. The results confirmed that TE-85 and Saos-2 had the highest levels of relative expression for COL1A2 compared to MG-63. ALP mRNA also showed its highest expression in Saos-2 and lowest in MG-63. OPG mRNA levels were similar in TE-85 and Saos-2, (2.5 and 2.6 fold respectively), and MG-63 expressed the lowest levels. This suggests that Saos-2 is the most mature; MG-63 is the least mature of the cell lines and TE-85 intermediate between the other two.

GIP-R has been described in chondrocytes, osteocytes, osteoblasts and in two of the cell lines studied here (MG-63 and Saos-2) [7,20], however the novelty of this current work is the profile of expression for those receptors which may be evident in different stages of maturity and correlated with the maturity of the cell lines. MG-63 and Saos-2 express GHS-R but no visual differences were demonstrated in earlier qualitative PCR screening [20]. Also GHS-R peaked three days after induction of osteoblastic differentiation of a murine cell line and the expression decreased with time, *i.e. *in more mature osteoblasts. In the current work GHS-R expression was greatest in the cell lines representing early osteoblasts (MG-63 and TE-85), while the most mature showed the lowest expression, in keeping with the time course of expression shown in the previous report. GHR has been shown to induce direct responses in bone cells through its receptor, in several different ways: differentiation, proliferation and/or viability [19-21].

GLP-1R had not previously been reported in human osteoblasts, or in osteoblastic cell lines like MG-63 or Saos-2 [7,12] but lately a study detected the presence of a functional receptor in a murine cell line, independent of the cAMP linked receptor [13] which is different from the human one we described in this paper. We indeed confirmed that Saos-2 cells do not express the receptor we looked for. However, TE-85 and MG-63 were positive for GLP-1R. The reasons for the discrepant results in MG-63 are not clear, but an explanation for it may lay in the size difference of the amplification template: smaller templates show more efficiency when they are amplified [34,35]. In the present work, the size of the template was much smaller (114 bp) than the size used in the previous report (695 bp), because qPCR calls for small templates. One study [12] showed that in a knockout animal model lacking GLP-1R, cortical BMD at tibia and lumbar spine was significantly reduced. They also established the lack of effects of GLP-1 treatment on Saos-2 cultures, which is in agreement with the current study demonstrating lack of GLP-1R.

The authors proposed that GLP-1R is essential in the control of bone resorption indirectly, since this receptor is expressed in thyroid C cells, and GLP-1 is able to stimulate calcitonin production in those cells with calcitonin receptor being able to inhibit osteoclastic bone resorption. No studies have confirmed GLP-2R presence either in osteoblastic or osteoclastic cells, and only two short reports mention its existence in bone-related cells [36,37], in contrast, the effects on bone resorption after administration of the peptide in clinical trials have been well documented [17,38,39]. GPR39 is believed to be the receptor for obestatin [22,40], but some reports have questioned that possibility [24,41,42]. A study demonstrated that transformed human embryonic kidney cells (293T) when transfected with a plasmid encoding human or mouse GPR39, but not GHS-R, exhibited high-affinity binding to an analogue of obestatin (monoiodobestatin) [26]. These receptors have not been described in bone-related cells, and the only record is listed in GenBank reporting its presence in a chondrosarcoma cell line (CA749039.1). In this current work PCR reactions showed positive signals for the receptor however the relationship between this receptor, obestatin and their influence on bone should be interpreted with caution.

Regarding functional responses, Saos-2 exhibited the most significant changes following exposure to GIP in terms of increases in cell viability, ALP secretion and P1NP production, according to the level of expression for this receptor. GHR and OB also induced significantly higher levels of ALP activity in Saos-2. In addition, it has been demonstrated that GIP and GHR, are able to induce functional responses in osteoblast-like cells [5], or to promote bone formation [19,21]. These observations are consistent with the findings in this study, showing responses at the concentrations from 10^-12 ^to 10^-9^M. This is relevant in relation to the physiological response *in vivo *as these concentrations are equivalent to the plasma levels that these hormones reach after meals [38,43,44]. However, a cautious note on this finding is the fact that a significant change in ALP activity is an indicator that a change in bone cell activity is taking place, and not only suggestive of bone formation as it has been demonstrated that increased values are associated with bone loss in aging, osteoporosis and menopause [45]. Levels of this enzyme decrease with therapy antiresorptive agents [46], but can increase again in response to anabolic treatments like teriparatide (recombinant form of PTH) [47].

MG-63 and TE-85, representing younger osteoblasts, showed significant higher levels of cell viability when the cell cultures were incubated in the presence of GLP-1 or GLP-2, as they showed higher expressions for these receptors. However they did not display any significant changes in terms of ALP secretion and TE-85 only responded to GHR at its highest concentration (10^-9^M). This lack of response might be attributable to lower ALP synthesis (as assessed by the relative level of ALP mRNA expression) rather than an absence of stimulation. No major changes were demonstrated for Saos-2, confirming the lack of the receptors in these cells.

Decreased P1NP concentrations were observed in supernatants from early osteoblasts-like (MG-63 and TE-85) after long exposure to GIP, GLP-1, GLP-2, OB in TE-85 and after GLP-1 in MG-63 cells. Short exposure did not cause significant changes and a sustained stimulation displayed a negative effect. The possibility that this pattern was caused because cell health was compromised can be rejected, as the viability assay did not show any significant cell death after long exposure to the peptides. Thus the changes in P1NP production, either by P1NP degradation or a decreased production of collagen, by the cell lines were caused by the presence of the peptides. There are no studies reporting changes in P1NP production by cells *in vitro *in response to these peptides and this is the first study to examine this bone marker in these cell lines. Conversely, Saos-2 cells showed significant increases of P1NP levels following GIP and OB. This is consistent with the studies that show GIP stimulates bone formation [5,9,48,49]. These opposite effects in response to GIP, increased levels in Saos-2 and decreased concentrations in TE-85, can be related to other findings which after prolonged exposure to high concentrations of GIP there was downregulation of the GIP-R in Saos-2 cells [5]. This finding may underpin the results observed in TE-85 and may account for the modest decrease of P1NP observed in MG-63 at the highest GIP concentration. On one hand GIP can promote bone formation (which is consistent with the results shown here for Saos-2 in terms of ALP and P1NP increases), but also a downregulation of the receptor might be a feasible explanation for the decreasing levels found in TE-85 after longer exposure to higher concentrations of the peptide.

It has been repeatedly reported that GLP-1R is not present in osteoblasts, but it should be noted that the published data refer mainly to the studies performed using Saos-2, which we confirmed do not express the receptor. TE-85 and MG-63 express the receptor and exhibited some significant responses to this peptide in the viability assay, and in P1NP secretion after long exposure to GLP-1. These observations support previous studies which show the role of GLP-1R in the mediation of bone resorption [12] and the possible association of GLP-1 treatment with the improvement in bone disorders linked to glucose intolerance [11]. While the reports linking GLP-1 to bone activity are few, there are more studies showing effects of GLP-2 in bone, and all of them found reductions in bone resorption markers, in clinical trials [17,38,39,50]. In the current work, the responses to GLP-2 were observed in TE-85 and MG-63, with significant decreased secretion of P1NP and OC. Most clinical trials have not shown any significant difference in the levels of those markers after treatment with GLP-2 [16,39,50]. Here, GLP-2 produced a significant response in TE-85 decreasing P1NP and in MG-63 caused a significant increase after 48 h and a significant decrease after 120 h. No regulation of the receptor has been suggested in previous reports.

GHR can induce responses from bone-related cells *in vitro *[19-21]. We confirmed these findings and a significant increase of ALP was found in Saos-2 and TE-85 but no significant changes were found at any time point for P1NP or OC. We regard that GHR may be an important modulator in the cells with a higher degree of differentiation, although the expression was more important in MG-63, these cells did not respond significantly to this peptide in any of the tests. In agreement with the latter experimental data, there is a clinical study reporting no significant effects of a GHR infusion on plasma CTX or P1NP when healthy and post-gastrectomy patients were studied [51] but an inverse relationship existed between baseline plasma GHR and CTX, suggesting that GHR may have a role regulating bone resorption.

OB caused significant ALP and P1NP increases in Saos-2, but TE-85 displayed decreased responses. The only data on OB effects is a short report where a human chondrocyte cell line, C28-I2, was studied and no changes were found in ALP production [52]. Some contradictory results have been reported where pancreatic beta cells displayed higher levels of survival after OB treatment [53] and no effect on cadiomyocytes viability was observed [54].

These paradoxical results, also observed in some of the clinical trials, may be explained by the nature of the receptor for these ligands which belong to the G protein coupled receptors and may be desensitised after long exposure to the ligand. In the case of GIP in Saos-2 a significant increase with the lowest concentration, might be explained by changes in receptor expression. Because the receptors for the hormones studied are GPCR, they can be subjected to some desensitization or rapid attenuation of receptor sensitivity after exposure to agonists [55].

## Conclusions

Given all the observations, it is tempting to hypothesise that osteoblastic cells respond to the gut peptides stimuli in feeding/fasting states depending on their stage of differentiation and on the duration of exposure to the gut hormones. In this way, the activity of bone cells is affected since the neuronal stimulus to eat is triggered, represented in this instance by GHR, and is under the influence of nutrients transiting through the gastrointestinal tract. The regulation of the gut receptors on bone could be another mechanism in the modulating bone metabolism. The behaviour of the osteoblastic cells in presence of peptides may vary with downregulation due to either sustained exposure or high circulating concentrations or both, since these receptors are GPCRs and they are able to decrease the responses to stimuli by several mechanisms that minimise the reaction to the ligand.

The ultimate goal of this research was to study the differential responses from the cell model, once the presence of receptors was assessed. However, further translational proteomics to explore the development through a primary cell osteoblastic development should be in order for prospective work.

In summary, we have shown that osteoblast-like cells express the receptors for five gut hormones, and they exhibit contrasting reactions when the correspondent ligands are present in the cell cultures.

## Competing interests

The authors declare that they have no competing interests.

## Authors' contributions

ELPP performed the design of primers and carried out the assays for the PCR, viability, ALP, P1NP and osteocalcin and statistical analysis. LRR, JAG and WDF contributed to the original idea, the design of the experiments, and draft the paper. PJMW helped to the cell culture, PCR and draft the paper. All authors read and approved the final manuscript.
